# Dietary Supplements Use among Athletes in Lebanon: Knowledge, Attitudes, Practices, and Correlates

**DOI:** 10.3390/foods11101521

**Published:** 2022-05-23

**Authors:** Zahra Sadek, Hala Mohsen, Saja Yazbek, Zein Al Abidin Nabulsi, Ahmad Rifai Sarraj, Maha Hoteit

**Affiliations:** 1 Laboratory of Motor System, Handicap and Rehabilitation (MOHAR), Faculty of Public Health, Lebanese University, Beirut 6573, Lebanon; zahrasadek81@hotmail.com (Z.S.); ahmadrifaisarraj@ul.edu.lb (A.R.S.); 2 PHENOL Research Group (Public HEalth Nutrition prOgram-Lebanon), Faculty of Public Health, Lebanese University, Beirut 6573, Lebanon; halamohsen17@hotmail.com; 3 Lebanese University Nutrition Surveillance Center (LUNSC), Lebanese Food Drugs and Chemical Administrations, Lebanese University, Beirut 6573, Lebanon; 4 Faculty of Public Health, Lebanese University, Beirut 6573, Lebanon; saja_yazbek99@hotmail.com (S.Y.); zeinisp1@gmail.com (Z.A.A.N.)

**Keywords:** dietary, supplements, knowledge, attitudes, practices, Lebanese, athletes

## Abstract

Athletes are under the utmost pressure to reach excellence in their performance and achieve the desired outcomes in competitions, prompting them to use dietary supplements. Given the threats to both health and eligibility, it is crucial to observe the prevalence, sources of information, knowledge, attitudes, and practices (KAPs) among Lebanese athletes practicing their sports for at least two years. In the present paper, a cross-sectional study is performed using the snowball sampling method, in which a self-administered KAP questionnaire is used to collect data from 455 athletes (mean age: 27.4 ± 7.9 years; men: 73.1%) participating in four sports categories (ball games, combat sports, endurance sports, and weightlifting). Among the Lebanese athletes, the prevalence of dietary supplement (DS) use was 74%, where half of them had predominately used sports supplements. Athletes in Lebanon heavily rely on coaches (74%) and online sources, including webpages and social media (64%), as key information sources for DSs. The findings suggest that significant proportions of athletes show knowledge deficits and unsatisfactory attitudes towards multiple aspects related to supplementation. Moreover, education and sports type modulate the use of DSs among athletes. Furthermore, 34% reported using supplements without a recommendation from specialists, and 69% admitted to not reading the supplement’s nutrition facts. This study urges the need for the regulation of concerned authorities and education programs to help overcome the existing challenges.

## 1. Introduction

Training and nutrition have a strong interplay in acclimating the body to build functional and metabolic adaptations [1]. Trained athletes walk a thin line between training hard and maximizing their performance [2], optimizing their nutrition, correcting nutritional deficiencies, increasing energy, increasing the rate of exercise recovery, improving their immune system, and maintaining health [3]. According to much evidence, well-planned nutrition strategies and personalized nutrition plans can enhance the performance of, and recovery from, sporting activities [1]. Thus, athletes’ nutrition plans should be adjusted to address the health; dietary demands; performance goals; physical features (i.e., body size, shape, growth, and composition); practical problems; and food preferences of individual athletes [1]. As a consequence, multiple published consensus statements on optimal food, hydration, and dietary supplements (DSs) have increased, to date, reflecting an increase in internationally endorsed dietary guidelines for athletes [4,5]. Dietary supplements, defined by the Dietary Supplement Health and Education Act of 1994, are “any product that is consumed for the purpose of supplementing the diet and may contain one or more dietary ingredients, including vitamins, minerals, herbs, amino acids, and other substances, intended to supplement the diet”, and may assist in ameliorating an individual’s sporting performance [1,6]. In addition, sports supplements defined as “products marketed for body-building, weight-loss, pre-workout/energy” have been laced with anabolic steroids and amphetamines [7,8,9]. Discussing the global sports supplement market, the significant internal and external pressures and the misuse of DSs led to high demand and availability on the market, despite insufficient supporting scientific evidence [10,11]. This has given rise to multi-billion dollar corporations that firmly market supplements as powerful ergogenic aids [12]. The safety of ergogenic aids and DSs has been frequently discussed in many reports. Eichner and Tygart (2016) reported that 14% to 18% of DSs contain illegal contaminants, and it should be noted that even higher percentages of muscle-building supplements may contain actual drugs [7]. In addition, according to solid evidences, the routine use of DSs is associated with an increment in athletes’ doping susceptibilities [13] and the ingestion of contaminated supplements has caused athletes to fail drug tests [14,15,16], which threatened public health. For instance, in 2013, 44 athletes were admitted to the Hawaii Department of Health for “acute onset hepatitis of unknown origin”, [17]. Among them, 82% had used the supplement OxyELITE Pro, which contained the stimulant 1,3-Dimethylamylamine, also referred to as DMAA. The consumption of this supplement led to the hospitalization of 14 persons, one death, and two persons requiring liver transplants [17]. Without a doubt, dietary knowledge affects attitudes and practices among athletes who often rely on their coaches for nutrition/DSs guidance [18]. Obviously, searching for proper nutrition through the internet and the use of DSs are growing, however, reliable and authentic information should be evaluated. This has the potential to cause harm if the coaches and athletes are misinformed [18]. Similar to several other places, the production, promotion, and marketing of DSs in Lebanon is unregulated, putting the athletes at risk in relation to unintended doping through the use of contaminated DSs. Therefore, we aim in this study to assess the knowledge, attitudes, and usage of DSs among Lebanese athletes who practice their sports for at least two years.

## 2. Materials and Methods

### 2.1. Study Design and Sampling Procedure

**Figure 1:**
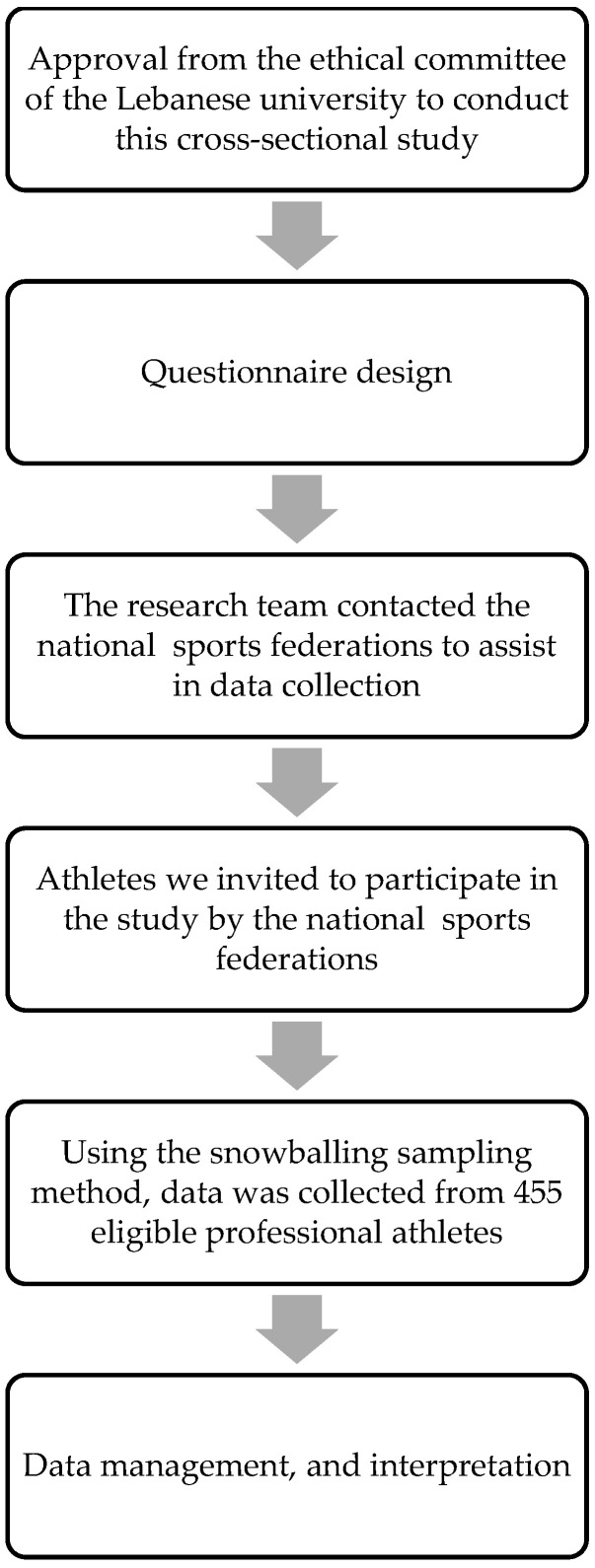
Flowchart of study design.

This investigation is a cross-sectional study conducted in Lebanon from February to August 2021, using the snowball sampling method. The athletes were asked to participate in the study by the national sports federation after exposing them to the study objectives. Eligible athletes who showed a willingness to participate in the study were invited to fill out an online form of a self-administered questionnaire via various social media platforms (i.e., WhatsApp, Facebook, Instagram, and LinkedIn). The athletes who participated in the study invited their teammates and other eligible athletes to take part in this study. The study design is illustrated in Figure 1.

### 2.2. Participants

For a power exceeding 80%, the Kish and Leslie formula [19] was used to include a sample size of 340 participants. A non-response rate of 10% was anticipated; thus, 455 athletes were included. A response of 100% was obtained. To be considered for inclusion, athletes had to be at least 15 years old, Lebanese, and officially belonging to the Lebanese sports federations, located in Beirut. All eligible athletes approved being enrolled in this study after being contacted. The sampled population included professional and amateur athletes who had been practicing their sports for at least 2 years. Of the 455 athletes, 73.1% were males, and 26.9% were females. Around 95% of the athletes were competing at the national level. On average, we recruited 455 athletes from 4 sports categories: 43.5% were participating in ball games (football, basketball, handball, futsal, beach soccer, tennis, table tennis, squash, and speedball); 27% in combat sports (taekwondo, karate, judo, boxing, wrestling, and mixed martial arts); 16.3% in endurance sports (running, swimming, and cycling); and 13.2% in weightlifting. The participants were collected through convenient sampling. The use of DSs was the dependent variable. The independent variables included participant demographic characteristics (age, sex, and level of education), duration and type of the sport practice, and the level of competition.

### 2.3. Study Instrument

A pre-tested self-administered questionnaire encompassed 3 main sections. The socio-demographic characteristics of the study participants were captured in the first section. It included age, gender, nationality, marital status, and education level. The second part covered the personal information of the athletes, including sports type, period being in sports (<5 or >5 years), time spent exercising in a week, and their competition level (international, amateur, first-class, third-class, or fourth-class players). In addition, athletes reported their body weight and height, allowing us to assess their weight status after obtaining the body mass index (BMI). We further classified athletes as underweight (BMI < 18.5), normal weight (BMI between 18.5 and 24.9), overweight (BMI between 25.0 and 29.9), and obese (BMI 30.0 and above). Note that the BMI is not a useful indicator of fatness among athletes [20,21], and it may overestimate their body fat [20,21]; however, it is still an affordable and easy-to-use method in research studies. The remaining parts of the questionnaire assessed the KAP related to dietary supplementation among the athletes. These parts were developed from similar studies [22,23,24] evaluating the nutritional knowledge (11 questions) of athletes and their attitudes (7 questions) and practices (6 questions) among DS use. The KAP part of the questionnaire was based on binary responses (Yes/No). The questionnaire underwent a preliminary analysis as part of a focus group on 20 athletes to assess the clarity and redundancy, and is then adjusted to present its final version. Additionally, the questionnaire was evaluated by experts in the field to make sure that the questions that were included covered all the aspects of the constructs being measured in the study. The reliability analysis of the KAP items indicated that all the variables that were measured were reliable, with Cronbach’s alpha values of 0.86 for the knowledge questions, 0.76 for attitudes questions, and 0.73 for practices questions. A Cronbach’s alpha of 0.7 and above was considered as acceptable.

### 2.4. Data Analysis

Statistical analysis was performed using the Statistical Package of Social Sciences Software (SPSS) (Version 21.0. IBM Corp: Armonk, NY, USA). Most study variables were categorical. The athletes’ ages were also categorized (youth (15–24 years old), and adults (>24 years old)). After evaluating the data’s normality, the variables were shown as frequencies and percentages. Chi-square was used to compare the different categorical variables. The binary logistic regression was applied to study the extent to which the significant variables from the bivariate analysis predict dietary supplementation among the athletes. A confidence interval of 95% was applied, and the level of significance was determined at 5% (*p* < 0.05 is considered significant).

### 2.5. Ethical Considerations

The study was approved by the Ethical Committee at the Lebanese University (#CU 25-2022), and it was carried out following the criteria approved by the Declaration of Helsinki. Written informed consent was provided by all participants and their proxy, when needed, before being enrolled in the study. The participation was voluntary with no penalties for denial or withdrawal.

## 3. Results

### 3.1. The Socio-Demographic and Personal Information of the Athletes

Overall, 455 athletes (mean age: 27.4 ± 7.97 years; males: 73.1%;) were enrolled in our study. Among them, 55.6% were adults (>24 years old), and the remaining athletes (44.4%) were youths (15–24 years old). Female athletes were younger than their male counterparts, with 50.8% of females being youths, compared to 42% of males, *p* < 0.001. Most of the athletes were single (66.8%) and 30.5% were married. Additionally, 39.1% were at a high school level or below, whereas the highest proportion of athletes (41.3%) had a bachelor’s degree from a university or were still undergraduates. Furthermore, 19.6% were postgraduates and had master’s or Ph.D. degrees.

In regard to the sports types, the highest proportion of athletes (43.5%) was ball-games players, and a salient proportion (27%) participated in combat sports. The remaining individuals were endurance-sports athletes (16.3%) and weightlifters (13.2%). More than half of the athletes (55.2%) were practicing their sports for more than 5 years, with a higher proportion among male athletes (59.7%), compared to females (42.6%), *p* < 0.001. Furthermore, the majority (80.9%) reported spending ≤ 10 h/week exercising, while the remaining (19.1%) reported exercising more (>10 h/week). In particular, male athletes appeared to exercise for a longer duration (>10 h/week) than females (23.1% vs. 8.1%, *p* < 0.001). Our sample population mostly included amateur athletes (54.4%), with the majority of female athletes (64%) being amateurs, *p* < 0.001. Moreover, the other athletes were international (11.8%) or first-class (17.8%) players. The remaining athletes were third-/fourth-class players (16%). In addition, most athletes (63%) were of a normal weight. Others were overweight (24.8%), obese (9.0%), and underweight (3.3%). The predominant proportion of female athletes (72.1%) appeared to have a normal body weight, *p* < 0.001. These findings are presented in Table 1.

### 3.2. Sources of Information on Dietary Supplementation

**Figure 2:**
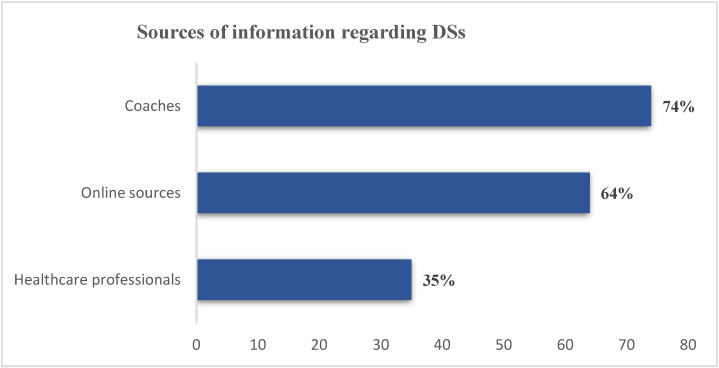
Sources of information regarding DSs.

There are numerous information sources concerning dietary supplementation. As shown in Figure 2, coaches were the prime sources of information (74%), followed by online sources (64%), including social media and websites. However, healthcare professionals were the least common source of information regarding DSs (35%) (Figure 2).

### 3.3. DS-Related Knowledge of Athletes

The responses of athletes to knowledge questions are shown in Table 2. It is evident that knowledge gaps were found in the areas related to the safety regulation of supplement products, where only 10.6% of participants agreed that the Food and Drug Administration (FDA) is responsible for taking action against contaminated DSs. Additionally, almost all athletes (93.1%) did not approve the statement that DSs should undergo safety tests before marketing. Moreover, 33.4% of the participants approved the following statement: “DSs may be replacers for a balanced diet”, assuming that supplement ingredients could substitute food nutrients. Furthermore, more than half of the athletes (55.2%) were non-knowledgeable about the possible supplement–drug interaction. Additionally, a considerable proportion (45.9%) was unaware that supplement products have health-related side effects. On the other hand, the majority (70.0%) concurred with the statement suggesting that DSs build and support muscles. ED-related knowledge was problematic, too, where 40% of athletes were not aware of the tendency of such drinks to energize the body. An appreciable proportion (39.3%) was mystified that EDs may cause insomnia, stress, and fatigue, and reported incorrect responses to these claims. Furthermore, salient proportions of athletes declined the statements that EDs may cause hallucinatory experiences and death (58.4% and 61.0%, respectively). No significant differences were observed between male and female athletes concerning their knowledge, except for the statement inquiring if DSs must be pretested for their safety before reaching consumers; almost all female athletes (98.3%) showed poor knowledge in relation to this question, compared to 91.3% of males, *p* = 0.045. Moreover, a higher proportion of male athletes (61.8%) refused the claim that EDs may cause hallucinations, in contrast to their female counterparts (49.1%), *p* = 0.023 (Table 2).

Thus, these results indicate that, from the total responses of athletes on the 11 knowledge-related questions, 53.2% were incorrect responses.

### 3.4. DSs-Related Attitudes of Athletes

The responses of the athletes to the attitude questions are shown in Table 2. Among all athletes, about half (53.8%) believed that sports supplements could improve their body shape. In addition, 42.4% agreed that sports supplements ameliorate athletes’ performances. Furthermore, 52.7% of them agreed that DSs could be used, even if food nutrients meet dietary needs. The majority (84.6%) reported feeling encouraged to use DSs if their teammates do so. Overall, 40.5% reported the use EDs because of their rich taste. At the same time, a salient proportion (55%) did not perceive the health-related risks associated with the consumption of energy drinks. In addition, 57.9% believed that sports supplements on the Lebanese market have probably been manipulated. Specifically, female athletes reported more unsatisfactory attitudes than their male counterparts towards the use of DSs to improve body shape (56.6% vs. 52.9%), ameliorate sports performance (50% vs. 46.2%), and imitate teammates’ behaviors (86% vs. 83.8%) (all *p*-values < 0.001). However, male athletes appeared to have more undesirable attitudes than female athletes toward the use of DSs, only if food nutrients are not sufficient to meet dietary needs (58% vs. 56.6%, *p* < 0.001) and regarding the possible manipulation of the DSs in the Lebanese market (47.1% vs. 43.4%, *p* = 0.004) (Table 2).

Subsequently, from the total responses of athletes on the 7 attitude-related questions, 55.7% were unsatisfactory responses, in which the practice of using dietary supplements in an attempt to imitate other teammates was the most alarming.

### 3.5. Prevalence of the DS Use and Supplementation Practices of Athletes

**Figure 3:**
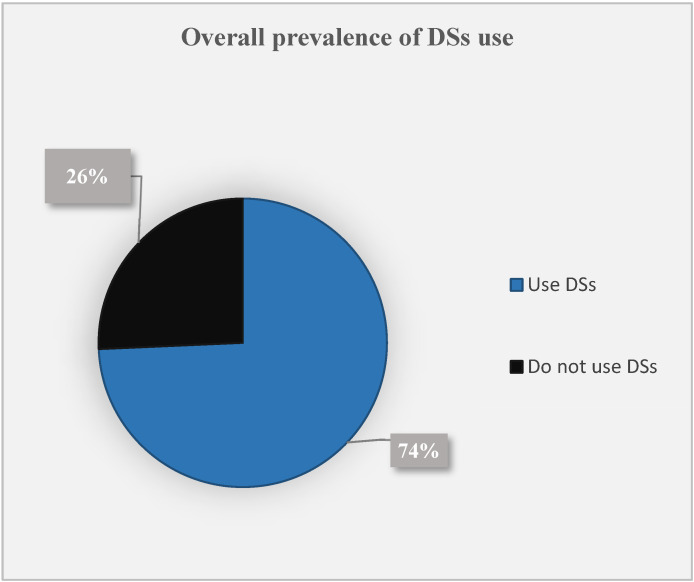
Overall prevalence of DSs use.

The overall prevalence of dietary supplementation was 74% in our sample population (Figure 3). The dietary supplementation practices of participants are presented in Table 2. About half (51.2%) reported using sports supplements, with no significant difference between the male and female athletes, in which 52.2% of male and 51.6% female participants reported the use of sports supplements, respectively, *p* = 0.462. In regard to vitamin and mineral supplements, the prevalent use of 35.6% was reported for the overall sample population. Similar proportions of male (34.5%) and female athletes (38.5%) admitted to the use of vitamin and mineral supplements, *p* = 0.408. Furthermore, 25.7% of athletes reported using EDs (males: 25.8%; females: 23.0%, *p* = 0.414). Among EDs users, 50% used to drink 1–2 cans per week, while the half remaining reported using 3–4 cans per week. The male athletes had a higher estimated use of EDs than their female counterparts, with 51.9% of males using 3–4 cans per week in contrast to 45.5% of females, *p* = 0.685. When asked about their reading practices of the supplements’ nutrition facts, 31% of athletes reported reading them, while the highest proportion (69%) reported not doing so. In addition, 34% claimed to use DSs without a recommendation from specialists, such as sports nutrition specialists. Hence, the latter findings suggest inappropriate supplementation practices among users (Table 2).

### 3.6. The Use of DSs According to Athletes’ Characteristics (Bivariate Analysis)

**Figure 4:**
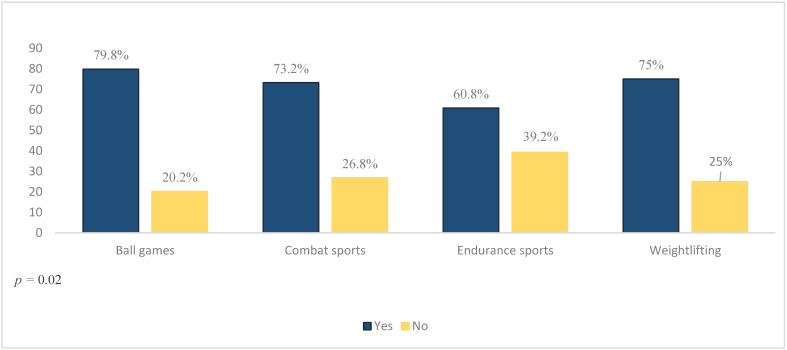
Overall prevalence of DSs use.

Table 3 shows the relationship between the use of DSs and athletes’ characteristics. The age of the athletes appeared to have no association with the use of DSs, with just an equal proportion of young (71.8%) and adult (76.2%) athletes reporting the use of supplements, *p* = 0.28. Additionally, the use of DSs did not differ significantly between male and female athletes, and more than 70% of both reported the use of supplementation (73.5% and 76.2%, respectively), *p* = 0.57. However, 78.7% of athletes with a university education level were supplements users, which was significantly higher than that reported by athletes at a high school level or below (67.4%), *p* = 0.01. Similarly, sports type appeared to be a predictor variable of DSs use; significantly, ball-sports athletes had the highest use (79.8%), followed by weightlifters (75%), combat-sports athletes (73.2%), and endurance-sports athletes (60.8%), *p* = 0.02 (Figure 4). Although international/first-class athletes had a predominant use of DSs (78.5%), the difference was not significant compared to amateur (72.8%) and third-/fourth-class (71.2%) athletes, *p* = 0.39. Moreover, just an equal proportion of athletes who were involved in their sport for 2–5 years (74.5%) and more than 5 years (74.1%) reported supplementation use, *p* = 0.92. Similarly, a close proportion of athletes who exercised ≤ 10 h/week (74.5%) and >10 h/week (73.6%) were supplements users, *p* = 0.86. Furthermore, more than half of DSs users were of a normal body weight (66.3%), followed by overweight (23.7%), obese (7.7%), and underweight (2.4%) athletes, *p* = 0.03 (Table 3).

### 3.7. Determinants of DSs Use among Athletes (Logistic Regression Analysis)

Based on the bivariate analysis, we attempted to determine the extent of the contribution of the variables of interest to the probability of dietary supplements use among athletes using the logistic regression analysis. Table 4 shows that adults had a 1.3-times-higher use of DSs than youths, although this finding was not significant (OR = 1.29, CI = 0.82–2.05, *p* = 0.27). Similarly, females had just a 9% higher probability to use DSs, compared to males (OR = 1.09, CI = 0.65–1.85, *p* = 0.74). On the other hand, holding a university degree significantly increased the probability of supplement use by 2 (OR = 1.81, CI = 1.17–2.79, *p* = 0.007). In regard to the sports types, ball-games athletes had 31%, 61%, and 30% higher probabilities of using dietary supplements, as opposed to combat-sports athletes (OR = 0.69, CI = 0.41–1.12, *p* = 0.17), endurance-sports athletes (OR = 0.39, CI = 0.21–0.67, *p* < 0.001), and weightlifters (OR = 0.70, CI = 0.35–1.40, *p* = 0.31), respectively. Furthermore, normal-weight athletes had the highest probability of using supplements, with around a 3-times-higher use than underweight athletes (OR = 2.65, CI = 0.87–8.05, *p* = 0.08) (Table 4).

## 4. Discussion

The current study assessed the use of dietary supplements among a sample of Lebanese athletes. In addition, it examined their knowledge and attitudes towards multiple aspects related to dietary supplementation. The determinants of dietary supplement use among athletes were also pointed out. Overall, coaches and online sources were the prime sources of information regarding DSs. The overall prevalence of dietary supplementation was 74%, with a predominant use of sports supplements (51.2%). Athletes’ education levels and sports types predicted the use of DSs.

In the current study, 74% of athletes reported that coaches were the prime sources of information regarding DSs, with the online sources coming next (64%), while a minority (35%) reported referring to healthcare professionals. Previous studies agreed with the latter finding, including that conducted among Malaysian athletes [25], who mostly referred to their coaches for information about dietary supplements. A systematic review of 53 studies addressing information sources about dietary supplements among athletes found that they often rely on coaches, trainers, friends, and family for information [26]. Early observations also found that the primary sources of nutrition information for athletes were trainers (40%) and coaches (24%), with a common belief that athletic trainers have good nutrition knowledge [27]. Interestingly, a study among adolescent athletes found that coaches are the primary source of information regarding supplements and their advice on protein supplement use was predominant [28]. In this regard, the correlates of coaches’ attitudes towards recommending sports nutrition products to their athletes suggested that subjective norm and perceived behavioral control were the key determinants [29]. However, the findings of the current study are in contrast to the results reported for a study conducted in Uganda, with the majority of athletes acquiring information on supplements from health professionals [12]. Additionally, internationally competing American athletes reported their preference to seek supplements information from a physician or nutritionist rather than other sources [26]. The available literature reveals that coaches appear to be a reliable source for supplements information in some countries, but not in others. This could be explained by the fact that coaches in middle- and high-income countries are often required to pursue supplemental coaching education; otherwise, they are not certified to practice their profession [12]. However, coaching certifications in most developing countries are not a strict requirement. [12]. Moreover, the competition level of the athletes also contributes to the information source preferences. Elite athletes and those competing at the international level avoids the seeking of information from coaches due to the introduced doping test and a fear of susceptibility to doping substances. It should be noted that the use of legal performance-enhancing dietary supplements increases the possibility of using illegal doping substances in later stages among athletes [13]. With that being said, athlete-based education interventions are imperative to promote a greater awareness of dietary supplements and doping agents [13].

The current study explored the idea that considerable proportions of the recruited athletes had apparent gaps in their knowledge related to supplementation, especially in areas related to the safety regulations, interactions, and side effects of supplement products. These findings complement a recent study that aimed to assess the knowledge of Lebanese people concerning dietary supplements, with appreciable proportions of participants having a shortage of knowledge and multiple misconceptions regarding DSs [30]. Furthermore, the latter results were corroborated by another study on Algerian athletes that showed a gap in the knowledge and risk perception of supplement use among 61% of them [31]. A research study among 874 high-performance British athletes showed a lot of inconsistencies between the motives for use and the type of supplements used, suggesting a lack of knowledge and understanding of supplementation effects and tendencies [32]. What makes the problem worse is the lack of regulations in the dietary supplement industry, with an abundance of supplement products containing prohibited ingredients. It has been reported that 5 to 20% of supplements contain prohibited substances, which are present either through inadvertent contamination or through deliberate adulteration during the production process [33]. Intentional contamination, with many substances being banned, continues to occur in dietary supplements sold in the United States, especially with muscle-building supplements [34]. Australian research found that 13 out 67 supplements contain anabolic drugs or stimulants, which are prohibited in sports supplements [35]. Additionally, 20% of nutritional supplements sold in Europe and the United States of America were reported to contain anabolic steroids [35]. The United States Dietary Supplement Health and Education Act (DSHEA) settled the regulatory frame for dietary supplements as foods through the FDA [36]. The FDA guides the supplement manufacturers in relation to the good practices for preparing, packing, labeling, and storing the supplements’ ingredients, to secure the purity, composition, and strength [36]. However, The FDA has the authority to regulate supplement products only after they become available in the marketplace, and supplement manufacturers do not need FDA approval before distributing and selling their products [37]. Moreover, we should not ignore the fact that athletes are over-pressed and deceived by the aggressive advertising of supplement products, which is specifically oriented towards them [38]. Athletes try to exploit every advantage to reach a competitive edge, and thus follow the fraudulent approaches of marketers, leading to knowledge problems, misconceptions, and inappropriate supplementation practices. In this regard, athletes should be in contact with physicians and dietitians to educate them about which supplements are banned, supplements’ efficacy, supplements’ adverse effects, and the possible interactions with drugs. It is recommended that dietary supplements be used based on the knowledge of qualified sports nutrition professionals [39]. As a rule of thumb, a dietary supplement could be used if there is a scientific rationale for a potential benefit for health or performance, has no adverse side effects, the athlete needs it to meet his/her dietary needs, and it has no long-term adverse effects [28]. A narrative review of the literature showed that education interventions had improved the knowledge of dietary supplements and doping agents among athletes. The education programs included group discussions about the side effects of substance use and the methods of doping; classroom sessions about the health, moral, social, and psychological aspects of nutritional supplements; and seminars about the doping-related medical aspects and athletic coaching [13].

Also of interest to the current study is the fact that the majority of athletes (84.6%) reported feeling motivated to use DSs when their teammates do so. In sports contexts, peer pressure does exist, especially among athletes in the adolescence stage of their lives. Both the perceived and actual pressures to adopt the interests of others in the team may lead to behavioral changes among athletes to foster uniformity and maintain a group identity [40]. “Everyone else is doing it” is a statement highlighting that athletes are at an increased risk of imitating teammates’ behaviors [41]. Thus, we suggest that dietary supplementation is possibly more prevalent in some sports teams than others, and in team sports than individual sports. A hypothesis that is further confirmed by the current study is that athletes involved in ball games (mostly team based) had a higher use of DSs than those participating in individual sports (combat sports, such as martial arts and weightlifting, and endurance sports, such as runners and cyclists). In the current study, 55% of athletes did not perceive the health-related risks associated with the consumption of EDs. In fact, in the United States, the energy drink industry amasses around USD 10 billion per year [42]. Energy drinks are often used to provide energy, stamina, athletic performance, and concentration [42]. The evidence-based data on the risks of energy drinks on health are abundant. The health risks associated with energy drinks are mainly related to their high sugar and caffeine contents [42]. The consumption of energy drinks is associated with anxiety and stress, hypertension, obesity, kidney damage, and stomach pain [42]. Among Lebanese people, 63.6% were found to consume energy drinks, with 29.6% of consumers experiencing at least one adverse effect, in which tachycardia was the predominant effect in 21.1% of cases [43]. Additionally, 42.4% of our participants believed that supplement products improved their performance, which is consistent with the other research findings showing that athletes use dietary supplements to shape their performance [12,22,44]. It is endemic among athletes to use mega doses of various vitamins to ameliorate their performance benefits [45]. Although research has shown that a vitamin deficiency impairs physical performance, vitamin supplementation for an athlete with an adequate diet has no advantageous impacts on their performance [45]. Furthermore, a review on the metabolic rationale of amino acids as nutritional aids in athletes found that, in contrast to their claims, branched-chain amino acids (BCAAs) do not enhance endurance performance, with a shred of weak evidence that glutamine supplements enhance immune function [46]. Moreover, some commercial supplements contain only little arginine and have a minor effect on growth hormone levels [46]. On the other hand, dietary supplements may still show performance improvements among athletes in certain circumstances. For instance, BCAA and arginine supplementation could improve performance in intermittent sprints on the second consecutive day of simulated handball games in well-trained athletes by potentially alleviating central fatigue [47]. Moreover, BCAAs, particularly leucine, have anabolic effects on protein metabolism by increasing the rate of protein synthesis and decreasing the rate of protein degradation in resting human muscles [48]. Additionally, creatine supplements may enhance performance during repeated short bursts of intense and intermittent activities, such as sprinting and weight lifting [49]. Supplementation with choline, an amine found naturally in food and grouped with B vitamins, increased plasma choline levels among marathon runners with a significant decline in the time required to run 20 miles [50]. Of note, there is a lengthy research history on the impacts of supplement products on performance, and all conclusions reached the same point showing that supplementation effects may differ from one athlete to another, between distinct exercises and due to a variety of other environmental factors. The use of any supplement product should be trailed before using it in a specific competition environment since, in worse scenarios, the deleterious effects may outweigh any expected benefits [51].

At the national level, it is somewhat difficult to compare our findings with the previous ones, due to the scarcity of data regarding the prevalence of dietary supplementation use among athletes. However, the prevalence of dietary supplementation in the current study (74%) is higher than that observed in 2017 among a sample of 611 sportsmen in Lebanon, where 23% of the participants used supplements products [52]. Moreover, it exceeds the prevalence use reported in 2010 among 512 exercises in gyms in Beirut city, where 36.3% of the enrolled participants were supplements users [24]. This discrepancy in the findings could be explained by the heterogeneity in the data collection and the characteristics of the targeted population. We noted that the previous studies included Lebanese sportsmen and exercisers from gyms across Lebanese governorates; thus, we assumed that our sample population included a higher proportion of athletes with sports professionalism, in which we included athletes from the official Lebanese sports federation, with about 30% being international or first-class players. It is well evident in the literature that elite and endurance-sports athletes rely more on dietary supplements than others with a lower profession to promote performance benefits [36,52,53]. We may also relate this difference to the COVID-19 pandemic, which recently emerged and propelled the general population, not only athletes, to rely more heavily on complementary and alternative medicine options, such as supplement products. In 2021, during the COVID-19 lockdown period, about 70% of the adult Lebanese population were using one or more supplement products with a common belief that DSs induce health and immunity-related benefits [30]. Although the prevalence of dietary supplementation in the present study is similar to that recently observed in the Lebanese population (70%) [30], the reasons for use and the supplements types differ between athletes and other population groups. In the present study, athletes showed a predominant use of sports supplements; however, about 70% of the Lebanese general population were using vitamin and mineral supplements [30]. Therefore, the general public often uses supplements for health-related reasons, unlike athletes who mostly supplement their diets to enhance their performance and recover faster [38]. In dietary supplementation contexts, athletes are considered as a distinctive subpopulation that relies heavily on protein-based dietary supplements than other subgroups [38].

In the Arab world, our observed prevalence of dietary supplementation is lower than, but close to, that reported among Saudi (93.3%) [22] (Table 5) and Algerian athletes (100%) [31] (Table 5), with almost all of them using one or more supplement product. In contrast, 48.9% of Egyptian athletes reported using any supplement products [54] (Table 5), indicating a lesser use of DSs than our sample population. This inconsistency could be partially explained by the difference in age characteristics between our study and that conducted in Egypt that included adolescents aged 13–18 years [54]. In Iran, a prevalence use of 45% was observed among collegiate athletes [55] (Table 5). Moreover, a cross-sectional international study showed that the prevalence of dietary supplementation among athletes from Serbia, Germany, Japan, and Croatia was about 82%, just close to our reported one [56] (Table 5). Furthermore, a similar prevalence use was observed among Australian (87.5%) [57], Canadian (87%) [58], and German (80%) [59] athletes (Table 5). However, athletes in the current study reported higher supplements use than Norwegian (53%) [60], Portuguese (66%) [61], Spanish (64% [62]; 58% [63]), British (62%) [64], Brazilian (45%) [65], and African (59%) [66] athletes (Table 5). In the United States of America, the prevalence of dietary supplementation was heterogeneous between different studies (26% [67]; 22.3% [68]; 61% [69]; 71% [70]; 98% [71]) (Table 5). Compared to Asian countries, a higher prevalence of dietary supplementation than that reported in the current study was observed in Korea (79–82%) [72], Singapore (77%) [73], and Sri Lanka (91.5%) [74] (Table 5). In summary, the overall prevalence of dietary supplement use in athletes is heterogeneous and differs from one study to another due to the differences in the sample size, age category, and different profession levels. In general, supplement use by athletes has an estimated range of 40 to 88% [75].

Overall, the highest proportion of our participants (51.2%) reported sports supplements use, followed by vitamin and mineral supplements (35.6%), and energy drinks (25.7%). These are in agreement with the previous findings for professional athletes in Saudi Arabia, which showed that 88.7%, 82.6%, and 52% of participants were consuming sports drinks, vitamin C, and multivitamins, respectively [22]. Furthermore, Algerian athletes showed an even higher use of dietary supplements, with athletes predominantly using gainers (25%), whey protein (20%), and branched-chain amino acids (BCAAs) (20%) [31]. Among German elite athletes, 80% reported using at least one supplement product, with minerals, vitamins, sports drinks, and energy drinks being the most commonly consumed [59]. Female athletes were observed to be the predominant users of iron supplements attempting to compensate for menses loss, which is exacerbated by hard training [76].

In the present study, only 31% of athletes reported reading the nutritional facts on the supplement products, with 34% taking the supplement products on their own without prescriptions from nutrition or health specialists. Dangerous supplementation practices among athletes, especially the elites, were highlighted by many researchers. Supplement products are often used with no clear understanding of their potential benefits and risks and without consultation from a sports nutrition professional [77]. It is frequently observed that athletes often use a combination of supplements and ignore the dosing recommendations [77]. Competitive and recreational athletes reported the overuse of supplement products in an attempt to support a poor-quality diet with a mistaken perception that an ordinary diet could not provide the necessary nutrients in adequate quantities [77]. This was further confirmed by the findings of the current study, with 52.7% of athletes agreeing that DSs could be used even if food nutrients meet the dietary needs. It was found that four out of five athletes were not familiar with platforms to check the safety and quality of supplement products, and they solely relied on the brand name of the product [62]. Furthermore, in many countries, athletes appeared to use more over-the-counter nutritional supplements, considering them legal, safe, and beneficial [78]. However, the sports supplement industry is not well regulated, with improper supplementation being a source of doping violations in multiple situations [78]. In addition, the supplements’ labels are not often tested, and they frequently contain unlisted substances [78]. As a result, health consequences can vary and may be lethal due to severe complications, including sudden cardiac death and arrhythmias, atherosclerosis and heart attacks, high blood pressure, heart failure, and blood clots [78].

The education status and sports type of the athletes were the predictor factors of dietary supplementation in the present study. In particular, having a university degree (vs. high school or below) increased the probability of supplement use by two. The education status of the athletes appeared to be a prime determinant of supplementation in the previous observations, with athletes with a higher education level (tertiary levels) appearing to rely more heavily on DSs than those with a lower education level [12,38,59]. Additionally, in the current study, ball-games athletes were more likely to use dietary supplements than combat-sports athletes (by 31%), endurance-sports athletes (by 61%), and weightlifters (by 30%). Sports types contributed to the supplementation patterns in multiple research studies. This data coincides with the study conducted on Ugandan athletes, where most supplement users were either basketball or rugby players [12]. The highly demanding schedule could be one possible explanation, in which ball-games players have more frequent competitions than athletes in different sports types. Thus, the winning desire, the desire to perform well in all competitions, and the hope to recover quicker may propel them to use dietary supplements more than other athletes. In the current study, the prevalence of dietary supplement use was also high among weightlifters (75%), possibly to achieve muscle and strength gains and increase adaptations to physical training and performance [79]. Moreover, combat-sports athletes and endurance-sports athletes also showed an appreciable prevalence of dietary supplement use (73.2% and 60.8%, respectively), which is still comparable to other sports categories. These findings reveal that sports types affect the extent of use of dietary supplements; however, dietary supplementation is a common practice among most athletes from all sports types and categories.

The age of the athletes did not predict DSs use in the current study. This data supported the previous observations [12,38,59]. However, some studies observed that adult athletes tend to follow unsupervised supplementation patterns more frequently than younger ones who usually use supplements with continuous monitoring by their parents and coaches [75]. Another interesting finding of the current study is that the gender of the athletes had no contribution to the use of dietary supplements, and this finding could be supported by that obtained in Uganda, with almost an equal proportion of male (12.7%) and female (15.4%) athletes reported to be using supplements [12]. A review of 159 studies [38] showed that, in general, the dietary supplementation prevalence is similar between male and female athletes. However, women appeared to use iron supplements more than men, with a larger proportion of active women appearing to be iron deficient compared to active men [38]. Thus, this leads athletic women to use iron supplements to enhance their health and performance. Moreover, in the current study, the time spent playing sports and exercising did not associate with supplement use. This is in contrast to the findings among Saudi gymnasium users [80], with 75.2% of supplement users being reported as exercising for more than 1 year.

### Strengths and Limitations

This observational study presented some limitations that should be taken into consideration to improve the applicability of our findings. First, reaching a causal inference was not possible due to the cross-sectional design of the study. Secondly, some data may not be accurate as the questionnaire was self-reported, and the estimated prevalence of dietary supplementation might be misreported, either intentionally or due to misunderstandings. Additionally, we collected no data on the nutrient adequacy of the athletes’ diets, which may be associated with supplement use among athletes. Furthermore, this study was conducted when Lebanon was in an enforced lockdown due to the COVID-19 pandemic, which may affect some of the study findings due to confounding effects. In addition, the questionnaire was not validated before, but its reliability was checked prior to the data collection and developed in referral to the instruments used in the preliminary studies [22,23,24]. Despite these limitations, nationally and in the Middle East, this study is the first to assess the knowledge, attitudes, and practices related to dietary supplementation among athletes affiliated with national federations. The authors believe that the current study could pave the way to control the unnecessary and inappropriate supplementation practices among athletes, with a hope to provide further research on this topic.

## 5. Conclusions

In conclusion, the findings of the current study indicate that dietary supplementation among Lebanese athletes is widespread. The reported prevalence of dietary supplement use in the current study is higher than what has been reported in other studies. Additionally, considerable proportions expressed a deficit in knowledge and critical misconceptions related to dietary supplementation. Online media and coaches were the most common sources of supplement information. Supplement use requires an individual assessment, followed by a recommendation from qualified sports nutrition professionals or other healthcare practitioners. The study’s findings urge the need for regulations and laws for concerned authorities and education programs targeting athletes to help overcome the existing challenges.

## Figures and Tables

**Table 1:** Socio-demographic and personal information of athletes.

		Overall N = 455		Males N = 333 (73.1%)		Females N = 122 (26.9%)		*p*-Value
		N	%	N	%	N	%	
Age in years	Youths (15–24)	202	44.4	140	42	62	50.8	<0.001
	Adults (>24)	253	55.6	193	58	60	49.2	
Marital status	Single	304	66.8	223	67.0	81	66.4	<0.001
	Married	139	30.5	101	30.3	38	31.1	
	Divorced	7	1.5	5	1.5	2	1.6	
	Widowed	5	1.1	4	1.2	1	0.8	
Education level	High school or below	178	39.1	147	44.3	31	25.4	<0.001
	Bachelor’s degree	188	41.3	129	38.7	59	48.4	
	Master’s or Ph.D. degrees	89	19.6	57	17.1	32	26.2	
Sports categories	Ball games	198	43.5	151	45.3	47	38.5	0.406
	Combat sports	123	27.0	91	27.4	32	26.2	
	Endurance sports	74	16.3	50	15.0	24	19.7	
	Weightlifting	60	13.2	41	12.3	19	15.6	
Duration being in their sports (years)	2–5	204	44.8	134	40.2	70	57.3	<0.001
	>5	251	55.2	199	59.7	52	42.6	
Time spent exercising (hours/week)	10 or below	368	80.9	256	76.8	112	91.7	<0.001
	More than 10	87	19.1	77	23.1	10	8.1	
Competition level	Amateur	248	54.4	169	50.7	78	64.0	<0.001
	International	54	11.8	42	12.6	12	9.8	
	First-class player	81	17.8	64	19.2	17	14.0	
	Third/fourth-class players	73	16.0	58	17.4	15	12.3	
Weight status	Underweight	15	3.3	5	1.5	10	8.2	<0.001
	Normal weight	286	63.0	198	59.5	88	72.1	
	Overweight	113	24.8	98	29.4	15	12.3	
	Obese	41	9.0	32	9.6	9	7.4	

**Table 2:** The athletes’ responses to the knowledge, attitudes, and practices (KAPs) questions.

DSs-Related Knowledge		Overall N = 455		Males N = 333		Females N = 122		
		N	%	N	%	N	%	*p*-Value
DSs may be replacements for a balanced diet.	No	303	66.6	222	66.6	81	66.4	0.956
	Yes	152	33.4	111	33.4	41	33.6	
DSs’ ingredients interact with those of drugs.	No	251	55.2	188	56.4	63	51.6	0.560
	Yes	204	44.8	145	43.6	59	48.4	
DSs have side effects on health.	No	209	45.9	155	46.5	54	44.3	0.665
	Yes	246	54.1	178	53.5	68	55.7	
DSs build and support muscles.	No	137	30.0	102	30.6	31	25.4	0.278
	Yes	311	70.0	231	69.4	91	74.6	
The FDA is responsible for taking action against adulterated DSs.	No	407	89.4	296	88.8	111	90.9	0.783
	Yes	48	10.6	37	11.2	11	9.1	
DSs should undergo safety tests before marketing.	No	424	93.1	304	91.3	120	98.3	0.045
	Yes	31	6.9	29	8.7	2	1.7	
EDs energize the body.	No	182	40.0	137	41.1	45	36.8	0.347
	Yes	273	60.0	196	58.9	77	63.2	
EDs cause stress and fatigue.	No	179	39.3	136	40.8	43	35.2	0.272
	Yes	276	60.7	197	59.2	79	64.8	
EDs cause insomnia.	No	179	39.3	136	40.8	43	35.2	0.223
	Yes	276	60.7	197	59.2	79	64.8	
EDs may cause hallucinatory experiences.	No	266	58.4	206	61.8	60	49.1	0.023
	Yes	189	41.6	127	38.2	62	50.9	
EDs overconsumption may cause death.	No	278	61.0	208	62.4	70	57.3	0.228
	Yes	177	39.0	125	37.6	52	42.7	
DS-related attitudes.		N	%	N	%	N	%	*p*-value
Sports supplements improve the body shape of athletes.	No	210	46.2	157	47.1	53	43.4	<0.001
	Yes	245	53.8	176	52.9	69	56.6	
Sports supplements ameliorate performance.	No	262	57.6	179	53.8	61	50	<0.001
	Yes	193	42.4	154	46.2	61	50	
DSs are necessary, only when food nutrients are not enough to meet dietary needs.	No	240	52.7	193	58	69	56.6	<0.001
	Yes	215	47.3	140	42	53	43.4	
I am encouraged to use supplements if my teammates do so.	No	70	15.4	54	16.2	17	14	<0.001
	Yes	385	84.6	279	83.8	105	86	
I use EDs because of their rich taste.	No	271	59.5	202	60.6	75	61.5	0.06
	Yes	184	40.5	131	39.4	47	38.5	
I believe that EDs have health-related risks.	No	250	55	185	55.5	68	55.7	0.07
	Yes	205	45	148	44.5	54	44.3	
Sports supplements on the Lebanese market have probably been manipulated.	No	192	42.1	157	47.1	53	43.4	0.004
	Yes	263	57.9	176	52.9	69	56.6	
DS-related practices.		N	%	N	%	N	%	*p*-value
Use of sports supplements.	No	222	48.8	159	47.8	63	48.4	0.462
	Yes	233	51.2	174	52.2	59	51.6	
Use of vitamin and mineral supplements.	No	293	64.4	218	65.5	75	61.5	0.408
	Yes	162	35.6	115	34.5	47	38.5	
Use of energy drinks (EDs).	No	338	74.3	244	73.2	94	77.0	0.414
	Yes	114	25.7	86	51.1	28	23.0	
Estimated use of EDs (cans/week) (among users; N = 114).	1–2	57	50.0	39	48.1	18	54.5	0.685
	3–4	57	50.0	42	51.9	15	45.5	
Read the nutrition label of DSs.	No	314	69.0	231	69.3	83	68.0	0.662
	Yes	141	31.0	101	30.0	39	32.0	
Use of DSs based on health specialists’ recommendations.	No	155	34.0	118	35.4	38	31.1	0.511
	Yes	300	66.0	215	64.6	84	68.9	

**Table 3:** The use of DSs according to athletes’ characteristics.

			DSs Use		
			Yes	No	*p*-Value
Age	Youths	N (%)	145 (71.8)	57 (28.2)	0.28
	Adults	N (%)	193 (76.2)	60 (23.8)	
Gender	Male	N (%)	245 (73.5)	88 (26.5)	0.57
	Female	N (%)	93 (76.2)	29 (23.8)	
Education level	High school or below	N (%)	120 (67.4)	58 (32.6)	0.01
	University	N (%)	218 (78.7)	59 (21.3)	
Sports categories	Ball games	N (%)	158 (79.8)	40 (20.2)	0.02
	Combat sports	N (%)	90 (73.1)	33 (26.9)	
	Endurance sports	N (%)	45 (60.8)	29 (39.2)	
	Weightlifting	N (%)	45 (75)	15 (25)	
Competition level	Amateur	N (%)	180 (72.8)	67 (27.2)	0.39
	International/ first class	N (%)	106 (78.5)	29 (21.5)	
	Third/fourth class	N (%)	52 (71.2)	21 (28.8)	
Duration being in their sports (years)	2–5	N (%)	152 (74.5)	52 (25.5)	0.92
	>5	N (%)	186 (74.1)	65 (25.9)	
Time spent exercising (h/week)	≤10	N (%)	274 (74.5)	94 (25.5)	0.86
	>10	N (%)	64 (73.6)	23 (26.4)	
BMI	Underweight	N (%)	8 (2.4)	7 (6)	0.03
	Normal	N (%)	224 (66.3)	62 (53)	
	Overweight	N (%)	80 (23.7)	33 (28.2)	
	Obese	N (%)	26 (7.7)	15 (12.8)	

**Table 4:** Determinants of DSs use among athletes.

Binary Logistic Regression Taking the DSs Use among Athletes (No (Reference) vs. Yes) as the Dependent Variable	Odds Ratio (95% Confidence Interval)	*p*-Valve
Age (reference: youth)	-	-
Adults	1.29 (0.82–2.05)	0.27
Gender (reference: male)	-	-
Female	1.09 (0.65–1.85)	0.74
Education (reference: high school or below)	-	-
Holding a university degree	1.81 (1.17–2.79)	0.007
Sports categories (reference: ball games)	-	
Combat sports	0.69 (0.41–1.12)	0.17
Endurance sports	0.39 (0.21–0.67)	<0.001
Weightlifting	0.70 (0.35–1.40)	0.31
BMI (reference: underweight)	-	-
Normal	2.65 (0.87–8.05)	0.08
Overweight	1.63 (0.49–5.37)	0.42
Obese	1.12 (0.31–4.01)	0.86

**Table 5:** An overview of the dietary supplementation patterns of athletes in different countries.

Country	Author(s) (Year)	Sample Size and Subpopulation	Sport Disciplines	Dietary Supplements	Mostly Consumed	Total Prevalence of DSs Use (%)
Middle East/North Africa region (MENA region)						
Saudi Arabia	Aljaloud, S. O. and Ibrahim, S. A. (2013) [22]	N = 105 professional male athletes (20 to 30 years old)	Football players from three teams residing in Riyadh: Al Hilal, Al Nasr, and Al-Shabab	Sports supplements, vitamins, minerals, carbohydrates, proteins, fish oils, herbals, and ergogenic aids	Sports supplements	93.3%
Algeria	Chabaiki, I. J. et al. (2020) [31]	N = 200 recreational and professional athletes (males: 95%; mostly 21–30 years old)	Body building, football, martial arts, athletics, cross fit, power lifting, and swimming	Mass gainers, whey protein, BCAA, glutamine, creatine, vitamins, fat burners, and arginine	Mass gainers	100%
Egypt	Tawfik, S. et al. (2016) [54]	N = 358 (13–18 years old; males: 56.4%)	Ball games (football, basketball, and volley ball); endurance (swimming, running, and cycling); weight class (wrestling, boxing, kickboxing, and weightlifting), and antigravity sports	Sports drinks, creatine, vitamins and minerals, and amino acids	Sports drinks and creatine	48.9%
Iran	Darvishi, L. et al. (2013) [55]	N = 192 male collegiate athletes; individual sports (mean age: 21.2 ± 2.2 years); team sports: (mean age: 22.1 ± 2.4 years)	Individual and team sports	Protein powders, amino acid powders, carbohydrates, slimming products, fish oils, ergogenic aids, creatine, caffeine, vitamins and minerals, glucosamine, and chondroitin sulphate	Multivitamins and vitamin C	45%
Asia region						
Korea	Kim, J. et al. (2011) [72]	N = 161 athletes; male: 89%; 14–37 years old)	Hockey, handball, basketball, badminton, table tennis, weight lifting, distance running, boxing, archery, taekwondo, judo, wrestling, gymnastics, and swimming	Vitamins, oriental supplements, amino acids, creatine, Korean ginseng, Korean red ginseng, and deer antler	Vitamins and oriental supplements	Males: 79%; Females: 82%
Singapore	Slater, G., Tan, B. and Teh, K. C. (2003) [73]	N = 160 (males: 53.1%)	Swimming/water polo, combat, hockey, rugby, sailing, racket sports, volleyball, netball, and sepak takraw	Sports drinks, caffeine, vitamin C, multivitamins/ mineral supplements, essence of chicken, birds nest, creatine, ginseng, and weight-gain powders	Sports drinks, caffeine, vitamin C, and multivitamins/ mineral supplements	77%
Sri Lanka	Rashani, SAN et al. (2021) [74]	N = 386 athletes (males: 66.8%; 18–41 years old)	Team (football, volleyball, rugby, hockey, kabaddi, and cricket); individual (wrestling, athletics, weight lifting, and karate); and mixed (wushu and badminton) sports	Multivitamin, electrolyte, protein, calcium, and creatine	Multivitamins	91.5%
Europe						
Spain	Baltazar-Martins, G., et al. (2019) [62]	N = 527 elite athletes participating in individual and team sports (males: 65.6%)	Bodybuilding, weightlifting, ball games, endurance sports, and combat sports	Proteins, amino acids/BCAAs, multivitamins, glutamine, creatine, carbohydrate powder, iron mix for recovery, joint support, omega 3, omega 6, magnesium, caffeine, B-alanine, and vitamin C	Proteins and amino acids/BCAAs	64%
Germany	Braun, H. et al. (2009) [59]	N = 164 elite young athletes (males: 47%; 16.6 ± 3.0 years of age)	Endurance, racquet, ball, combat, and other sports	Vitamins, minerals, carbohydrate, protein, and fat supplements; sports drinks; and other ergogenic aids	Minerals, vitamins, sports drinks, energy drinks, and carbohydrates	80%
Serbia, Germany, Japan and Croatia	Jovanov, P. et al. (2019) [56]	N = 348 athletes (males: 60.6%; 15–18 year olds) competing at the international level	Kayaking, rowing, canoeing, basketball, volleyball, swimming, athletics, boxing, soccer, tennis, karate, handball, water polo, dance, golf, weightlifting, archery, and fencing	Protein, carbohydrates, creatine, caffeine, NO reactor, beta alanine, glutamine, amino acids, vitamins and minerals, energy drinks	Protein supplements	82.2%
Norway	Sundgot-Borgen, J., Berglund, B. and Torstveit, M. K. (2003). [60]	N = 1620 elite athletes (males: 69%; mean age: 23.2 ± 4.7 years for males; mean age: 21.4±4.6 years for females)	NA	Vitamins, minerals, omega 3, antioxidants, ginseng, amino acids, creatine, and energy supplements	Energy supplements	53%
Portugal	Sousa, M. et al. (2013) [61]	N = 292 (males: 68%; 12–37 years old)	Volleyball, swimming, triathlon, cycling, judo, athletics, baseball, handball, rugby, gymnastics, basketball, fencing, and boxing	Multivitamins/minerals, sports drinks, magnesium, protein, glutamine, iron, sport gels, vitamin C, creatine, and antioxidants	Multivitamins/minerals, sports drinks, and magnesium	66%
Spain	Schroder, H. et al. (2002) [63]	N = 55 professional athletes (mean age: 25.1 ± 4.0 years)	Basketball	Multivitamins and minerals, sports drinks, miscellaneous supplements, amino acids, proteins, and carbohydrates	Multivitamins and minerals	58%
United Kingdom	Nieper, A. (2005) [64]	N = 32 athletes competing at the 2004 World Junior Championship (males: 62.5%; 18 years old)	Track and field	Ergogenic aids (creatine and caffeine) and recovery nutrients (vitamins/minerals, glucosamine, and glutamine)	Multivitamins and minerals	62%
South and North America						
United States	Barrack, M. T. et al. (2022) [67]	N = 2113 pre-adolescent endurance runners (males: 59.4%; mean age: 13.2 ± 0.9 years)	Track and field (running)	Vitamin/mineral supplements (multivitamins, vitamins C,D,E,B-complex, and others); non-vitamin/mineral supplements (amino acids, probiotics, diet pills, creatine, glutamine, herbs or botanicals); and sports foods (protein bars or drinks, electrolyte drinks, and energy bars)	Multivitamins	26%
United States	Ziegler, P. J., Nelson, J. A. and Jonnalagadda, S. S. (2003) [70]	N = 124 athletes (males: 34.3%; mean age for males: 16.9 ± 0.3 years; mean age for females: 15.2 ± 0.2 years)	Figure skating	Multivitamins, minerals, protein powders, amino acid powders, protein bars, energy drinks, energy bars, creatine, herbal supplements, and others	Multivitamins and minerals	71%
United States	Scofield, D. E. and Unruh, S. (2006) [68]	N = 139 adolescent athletes (males: 71%; mean age 15.8 ± 1.19 years)	Football, volleyball, basketball, wrestling, track and field, soccer, baseball, softball, power lifting, tennis, golf, cross country, swimming, and multisport	Creatine, MRP and protein, vitamins and minerals, diet energy	MRP protein	22.3%
United States	Brill, J. B. and Keane, M. W. (1994) [71]	N = 309 (males: 68%; 13 to 70 years old)	Bodybuilding	Vitamins, protein powder, amino acids, minerals, weight-gain formulas, carbohydrate formulas, anabolic supplements, energy boosters, fat burners, human GH releasers, liver supplements, and sterols	Vitamins, minerals, amino acids, and protein powders	98%
United States	Froiland, K. et al. (2004) [69]	N = 370 athletes (females: 55.8%)	Baseball, softball, volleyball, tennis, football, wrestling, bowling, yell squad and dance team, basketball, soccer, gymnastics, golf, track and field, swimming and diving, and rifle	Protein supplements, weight gainers, vitamin supplements, mineral supplements, herbals, and other supplements	Energy drinks	61%
Canada	Lun, V. et al. (2012) [58]	N = 440 athletes (men: 37%; mean age: 9.99 ± 5.20 years)	Soccer, ice hockey, Taekwondo, speed skating, volleyball, figure skating, athletics, alpine skiing, luge, and basketball	Sports supplements, multivitamins, minerals, carbohydrate sports bars, protein powder, meal-replacement products, vitamin c, ginseng, protein bar, sports gel, iron, essential fatty acids, calcium, echinacea, L-glutamine, and energy drinks	Sports drinks, multivitamins and minerals, carbohydrate sports bars, protein powder, and meal-replacement products	87%
Brazil	Nabuco, H. C. et al. (2017) [65]	N = 182 athletes (14 to 59 years old; males: 83%)	Endurance (triathlon, cycling, and swimming); bodybuilding; intermittent (volleyball, soccer, futsal, beach volleyball, tennis, and American football); combat (Taekwondo, karate, judo, kung Fu, Jiu Jitsu, MMA, boxing, and Mauy Thai); and other (athletics and shooting) sports	NA	NA	45%
South and East Africa						
Uganda	Muwonge. H, et al. (2017) [12]	N = 359 athletes (males: 74.7%; 15–35 years old)	Football, volleyball, rugby, netball, basketball, boxing, athletics, and cycling	Carbohydrate supplements, energy drinks, vitamin and mineral supplements, fish oils, protein supplements, herbal supplements, and ergogenic aids	Carbohydrate supplements, energy drinks, vitamin and mineral supplements, fish oils, and protein supplements	13.4%
South Africa	Janse van Rensburg, D. C. et al. (2018) [66]	N = 2550 amateur athletes (25 to 45 years old; 75% males)	Cycling	NA	NA	59%
Australia and Oceania						
Australia	Dascombe, B. J. et al. (2010) [57]	N = 72 (21.9 ± 3.9 years old; males: 50%)	Kayaking, field hockey, rowing, water polo, swimming, athletics, and netball	Vitamins, minerals, glucosamine, iron, caffeine, creatine, mixed proteins CHO, proteins, and others	Minerals and vitamins	87.5%

## Data Availability

Data is contained within the article.
